# Evaluation of Clinical Course of Gamma (P.1) Variant of Concern versus Lineages in Hospitalized Patients with COVID-19 in a Reference Center in Brazil

**DOI:** 10.4269/ajtmh.21-1264

**Published:** 2022-06-27

**Authors:** Alexandre P. Zavascki, Tarsila Vieceli, Priscila Lamb Wink, Fabiana Caroline Zempulski Volpato, Francielle Liz Monteiro, Julia Biz Willig, Charles Francisco Ferreira, Beatriz Arns, Guilherme Oliveira Magalhães Costa, Matheus de Souza Niches, Andreza Francisco Martins, Afonso Luís Barth

**Affiliations:** ^1^ Infectious Diseases Service, Hospital de Clínicas de Porto Alegre, Brazil;; ^2^ Internal Medicine Department, Universidade Federal do Rio Grande do Sul, Brazil;; ^3^ LABRESIS—Laboratório de Pesquisa em Resistência Bacteriana, Hospital de Clínicas de Porto Alegre, Porto Alegre, Brazil;; ^4^ Laboratório de Diagnóstico de SARS-CoV-2, Hospital de Clínicas de Porto Alegre, Porto Alegre, Brazil;; ^5^ Programa de Pós-Graduação em Ciências Farmacêuticas, Universidade Federal do Rio Grande do Sul, Porto Alegre, Brazil;; ^6^ Programa de Pós-Graduação em Ciências Médicas, Universidade Federal do Rio Grande do Sul, Porto Alegre, Brazil;; ^7^ Laboratório de Diagnóstico Molecular, Universidade Franciscana, Santa Maria, Brazil;; ^8^ Faculdade de Medicina, Universidade Federal do Rio Grande do Sul, Porto Alegre, Brazil;; ^9^ Bioinformatic Core, Hospital de Clínicas de Porto Alegre, Porto Alegre, Brazil;; ^10^ Departamento de Microbiologia, Imunologia e Parasitologia, Instituto de Ciências Básicas, Universidade Federal do Rio Grande do Sul, Porto Alegre, Brazil

## Abstract

The SARS-CoV-2 variant of concern (VOC) gamma (P.1) has increased transmissibility and resulted in elevated hospitalization and mortality rates in Brazil. We investigated the clinical course of COVID-19 caused by gamma and non-VOCs at a reference hospital in Brazil in a retrospective cohort study with nonelderly hospitalized patients from two periods, before and after the emergence of gamma. Cohort 1 included patients from both periods whose samples would be eligible for whole-genome sequencing (WGS). Cohort 2 was composed of randomly selected patients from Cohort 1 whose samples were submitted to WGS. A total of 433 patients composed Cohort 1: 259 from the first and 174 from the second period. Baseline characteristics were similar, except for a higher incidence of severe distress respiratory syndrome at admission in patients from the second period. Patients from the second period had significantly higher incidence rates of advanced respiratory support (adjusted hazard ratio [aHR]: 2.04; 95% confidence interval [CI], 1.60–2.59), invasive ventilatory support (aHR: 2.72; 95% CI: 2.05–3.62), and 28-day mortality from the onset of symptoms (aHR: 2.62; 95% CI: 1.46–4.72). A total of 86 (43 gamma and 43 non-gamma) patients composed Cohort 2. Patients with confirmed gamma VOC infections had higher advanced ventilatory support and mortality rates than non–gamma-infected patients. Our study suggests that non-elderly patients hospitalized for COVID-19 in the second period (used as a proxy of gamma infection) had a more severe clinical course. This might have contributed to higher hospitalization and death rates observed in the second wave in Brazil.

## INTRODUCTION

Since September 2020, four SARS-CoV-2 Variants of Concern (VOC) have emerged in different parts of the world, further complicating the efforts to control the COVID-19 pandemic.^1^ Alpha (B.1.1.7), gamma (P.1), and delta (B.1.617.2) are variants of concern (VOCs) associated with increased transmissibility and are implicated in COVID-19 surges followed by increased hospitalizations, intensive care unit (ICU) overload, and higher death rates.^1–4^ However, it has is not been determined whether these higher hospitalization and mortality rates may also be a consequence of increased severity of these VOCs or only due to the huge number of new infections. Distinct findings on the impact of VOC severity have been observed in previous populational studies addressing the influence of VOCs on hospitalizations and mortality.^4–13^

The gamma variant has caused a dramatic surge of COVID-19 cases, hospitalizations, ICU demand, and deaths in the Amazon region of Brazil.^3^ Gamma lineage has further spread to other Brazilian regions, leading to huge hospital system overload,^14^ and further spread to other South and North American countries^15^ and Europe.^16^

Although omicron (B.1.1.529) has recently dominated the epidemiological scenario worldwide,^17^ lessons taken from clinical characteristics of other VOCs are relevant to understand and estimate the public health impact of the emergence of other emerging VOCs, which may occasionally share similar features. In this study, we investigate the clinical course of COVID-19 caused by gamma and non-gamma SARS-CoV-2 lineages in nonelderly patients hospitalized at a COVID-19 reference center in Brazil.

## METHODS

### Study design, setting, and participants.

This is a retrospective cohort study carried out at Hospital de Clínicas de Porto Alegre (HCPA), a tertiary-care, COVID-19 reference hospital, located at the city of Porto Alegre (1,488,000 inhabitants), the capital of Rio Grande do Sul State, Brazil.

The study was composed by two cohorts. Cohort 1 included ≥ 18 and ≤ 65 years patients admitted at the emergency department of HCPA in two periods, classified according to the first detection of gamma variant in the state: first period, before the detection of gamma (June 1 to December 31, 2020) and second period, after the detection of gamma (February 1 to May 31, 2021). Because the first detection of gamma in our state was in January 2021,^14^ it was considered a month of transition from the predominance of other lineages to predominance of gamma, and patients admitted in this month were not considered for the study. Only patients whose real-time reverse transcriptase polymerase chain reaction (RT-qPCR) cycle threshold (Ct) value for both SARS-CoV-2 nucleocapsid protein N1 and N2 gene targets were < 26, which would more likely result in a high-quality whole genome sequencing (WGS), were considered eligible for the study. Patients were excluded if they were transferred from another hospital or if they were admitted because of non–COVID-19 related diseases and had a positive screening test. Cohort 2 was composed by a random selection of patients from Cohort 1. These randomly selected patients had their RT-qPCR samples submitted to WGS. The predefined number of the Cohort 2 was 86 (see statistical analysis later in the article). WGS was performed in a set of 10 to 20 samples. Patients were excluded if WGS generated a low-quality sequence and further excluded if the last set of sequenced samples outweighed the predefined number of 86.

### Ethical aspects.

This study is part of a research project on the epidemiology of SARS-CoV-2. It has been approved by the institutional ethics committee (project no. 2020-0163), which waived informed consent.

### Baseline characteristics.

The following variables were assessed at hospital admission: age, sex, body mass index, Charlson’s Comorbidity Index score^18^ and specific comorbidities, number of days from onset of symptoms to hospital admission, 6-point ordinal scale classification at admission,^19^ and partial pressure of arterial oxygen/ fraction of inspired oxygen (PaO_2_/FiO_2_) ratio.

### Outcomes.

The primary outcome was incidence of need for advanced respiratory support within 28 days from the onset of symptoms. Advanced respiratory support was defined as the requirement of supplementary oxygen by non-invasive ventilation, high-flow nasal cannula, mechanical ventilation, or extracorporeal membrane oxygenation. The institutional protocol recommends the use of noninvasive ventilation or high-flow nasal cannula for patients with peripheral oxygen saturation < 93% with low-flow oxygen supplementation > 5 L/minute, and with PaO_2_/FiO_2_ of 200 to 300 or > 24 respiratory movements per minute. The main prespecified secondary outcomes, which were evaluated in both cohorts, were incidence of need of invasive ventilatory support (requirement of mechanical ventilation or extracorporeal membrane oxygenation) from the onset of symptoms; 28-day mortality from onset of symptoms and from hospital admission. Other secondary outcomes (definitions are described in the supplementary methods), evaluated only in Cohort 2, were progression in an ordinal clinical scale of COVID-19 severity during the first 28 days, PaO_2_/FiO_2_ ratio during hospitalization, days alive and free of supplemental oxygen support, need of admission at an ICU, occurrence of documented deep venous thrombosis or pulmonary embolism, need of renal replacement therapy, need of prone positioning, and in-hospital mortality. A set of laboratory parameters was also evaluated in patients from Cohort 2 during the hospitalization, including C reactive protein, creatinine, D-dimers, leukocytes (total count and differential cell counts), and platelets.

### Molecular tests.

PCR and WGS procedures are described in the supplemental methods.

### Statistical analysis.

A sample size was estimated for Cohort 2 due to limited WGS test available, and it resulted in 86 patients (43 gamma and 43 non-gamma patients) as detailed in the supplemental methods. All patients eligible for sequencing were analyzed in the Cohort 1 as detailed earlier.

Bivariate analysis of gamma- and non-gamma-infected patients baseline characteristics was performed using Student’s *t*-test or Mann-Whitney tests for continuous variables and χ^2^ test or Fisher’s exact test for categorical variables.

Kaplan-Meier estimates for the need for advanced respiratory support were calculated, and the difference between groups was compared using the log-rank test.

Cox proportional hazard models were performed to evaluate the effect of gamma infection on the need of advanced respiratory support, need of invasive ventilatory support, and 28-day mortality from onset of symptoms including the following variables: age, sex, body mass index, and Charlson’s comorbidity score. Other baseline variables (PaO_2_/FiO_2_ ratio and clinical severity scale) were not considered in this model because they lied on a causal pathway between the exposure and the outcome and therefore are not confounders but intermediated variables. For 28-day mortality from hospitalization, other variables at admission were considered as detailed in the supplementary methods.

Other secondary outcomes were assessed as detailed in the supplemental methods.

The database was double entered, revised, and validated in the SPSS program (version 18.0). All tests were two-tailed, and a *P* value < 0.05 was considered statistically significant.

## RESULTS

### Patient characteristics.

**Figure 1.**
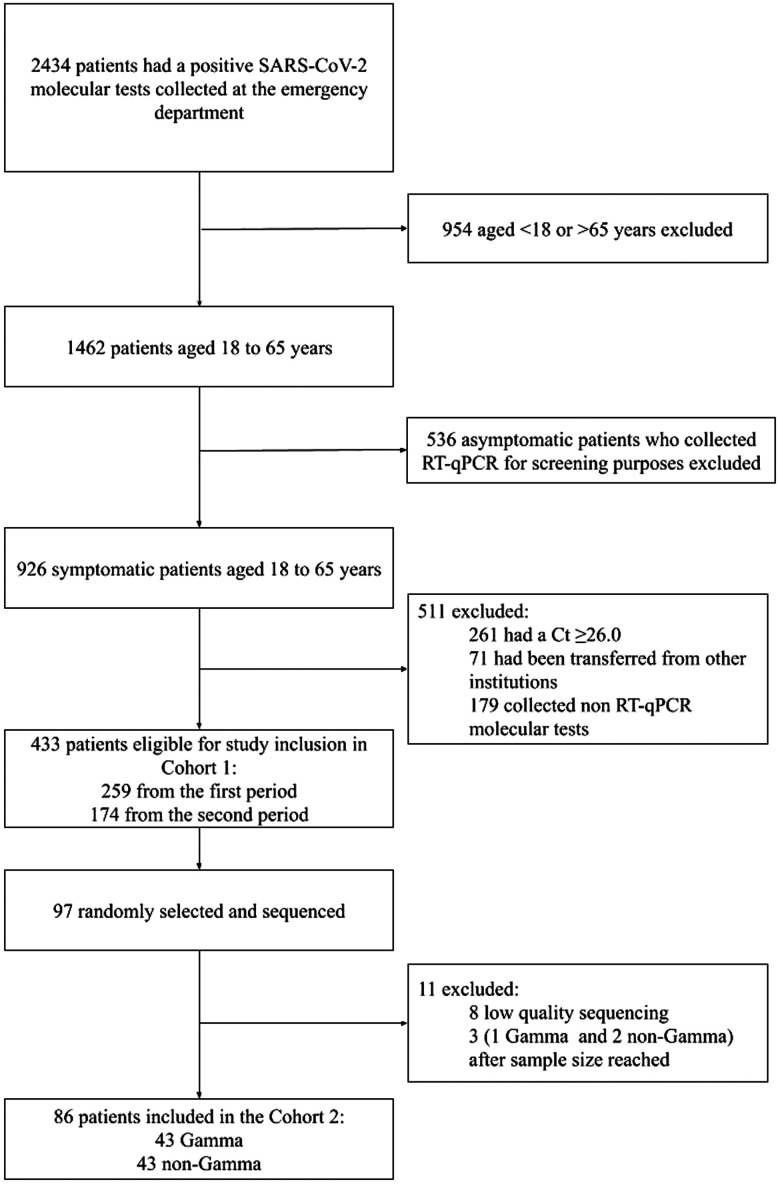
Study flowchart. Cohort 1: First period: June 1 to December 31, 2020 (before the first detection of gamma in Rio Grande do Sul State, where the study was conducted); and second period: February 1 to May 31, 2021 (after the first detection of gamma in late January 2021).

A total of 2,434 patients had a positive result of SARS-CoV-2 RT-qPCR from samples collected at the emergency department. Exclusions occur as displayed in the study flowchart (Figure 1). A total of 433 patients eligible for the study and composed the Cohort 1: 174 (40.2%) patients from the first period and 259 (59.8%) from the second period. Ninety-seven randomly selected patients had their samples sequenced, eight were excluded because the WGS presented low-quality (average coverage < 350), and one gamma patient recovered in the first period and two non-gamma from the second period were excluded because the first 86 patients planned to compose the Cohort 2 had already been included (these patients were analyzed only in Cohort 1).

Four of 43 samples from the gamma group were gamma sublineages (2 P.1.1 and 2 P.1.2). Most lineages included in the non-gamma group were B.1.1.28 (15, 34.9%) and B.1.1.161 (10, 23.3%); other non-gamma lineages are presented in the supplementary Table 1.

### Baseline characteristics.

In Cohort 1, patients from the second period had significantly lower comorbidity score than patients from the first period. Patients from the second period had significantly more severe acute respiratory distress syndrome and less frequently presented PaO_2_/FiO_2_ > 300 at admission and had significantly higher ordinal clinical scale scores at admission than patients from the first period (Table 1).

**Table 1:** Baseline characteristics of patients in Cohort 1

Characteristics	Second period (*N* = 174)*	First period (*N* = 259)*	*P*
Gender, male	89 (51.1)	136 (52.5)	0.86
Age, years	50.0 (40.0–58.0)	53.0 (43.0–60.0)	0.12
Charlson’s Comorbidity Index score	0 (0–1)	0 (0–2)	0.001
BMI, kg/m^2^†	31.2 (27.4–38.0)	31.0 (27.3–35.9)	0.44
BMI ≥ 30 kg/m^2^†	94 (58.4) [161]	140 (58.3) [240]	0.99
Time from onset of symptoms to hospital admission, days	7.0 (5.0–9.0)	7.0 (5.0–9.0)	0.52
PaO_2_/FiO_2_ at admission			< 0.001
> 300	55 (31.6)	156 (60.2)	
300–201	25 (14.4)	34 (13.1)	
200 101	47 (27.0)	48 (18.5)	
≤ 100	47 (27.0)	21 (8.1)	
Score on six-level ordinal scale			< 0.001
2: hospitalization without supplemental oxygen	30 (17.2)	115 (44.4)	
3: hospitalization with supplemental oxygen	82 (47.1)	108 (41.7)	
4: hospitalization with noninvasive ventilation or high-flow supplemental oxygen	39 (22.4)	22 (8.5)	
5: hospitalization with invasive mechanical ventilation and/or extracorporeal membrane oxygenation	23 (13.2)	14 (5.4)	

BMI = body mass index; PaO_2_/FiO_2_ = partial pressure of arterial oxygen /fractional inspired oxygen; Data expressed as *n* (%), median (interquartile range) or mean ± SD.

* Infections in patients from the second period are presumably caused by gamma and from the first period are presumably caused by non-gamma lineages.

† Thirteen (7.4%) patients from the second and 19 (7.3%) from the first period did not have BMI recorded.

In Cohort 2, gamma patients had a lower Charlson comorbidity score and lower platelet count at admission ( Supplemental Table 3). A total of 41 (95.3%) and 39 (90.7%) gamma- and non-gamma-infected patients (*P* = 0.67), respectively, received corticosteroids as a part of COVID-19 management during hospitalization. No other therapy such as remdesivir or tocilizumab was administered.

### Outcomes.

**Figure 2.**
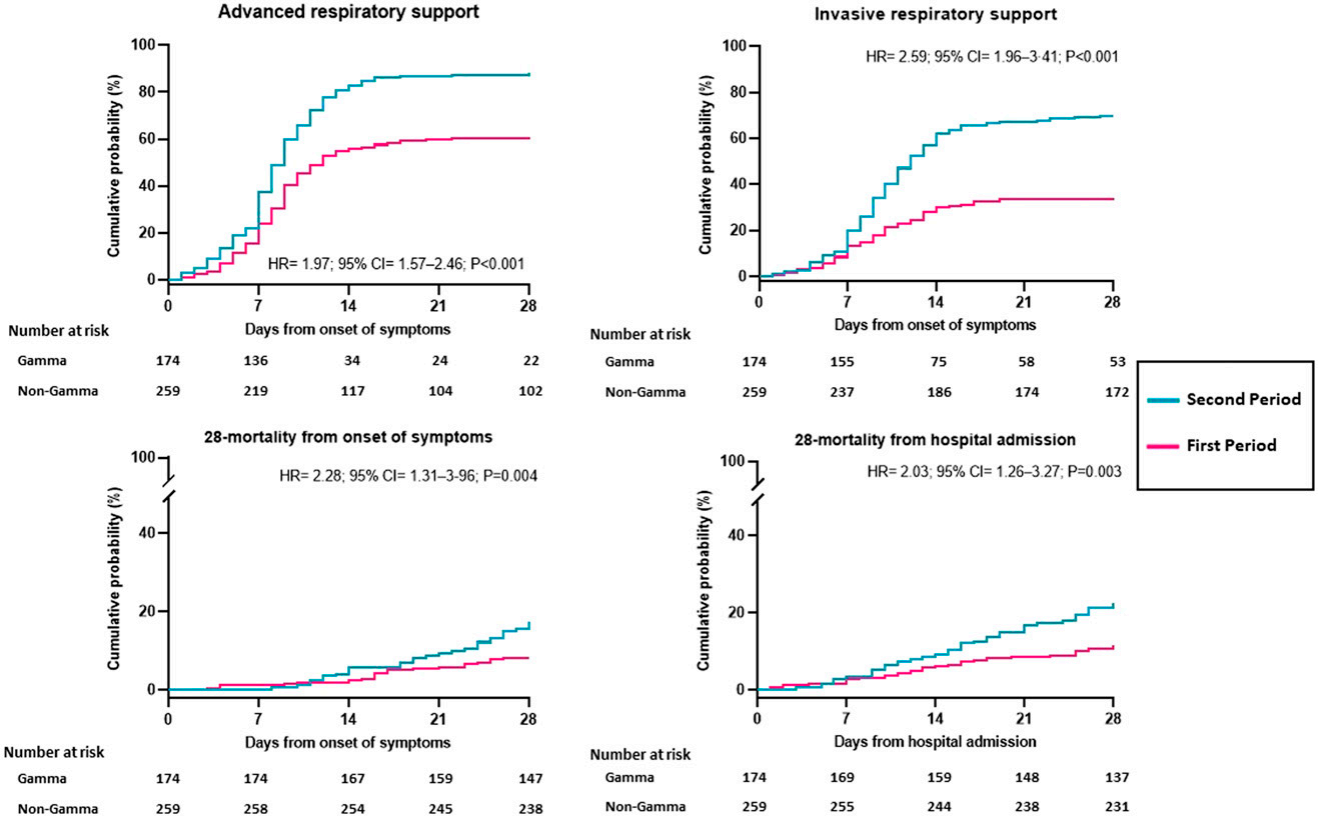
Primary and major secondary outcomes in the Cohort 1. A total of 153 (87.9%) of 174 patients from the second and 157 (60.6%) of 259 patients from the first period required advanced respiratory support; 121 (69.5%) and 89 (34.4%) from the second and first periods, respectively, required invasive respiratory support; 31 (17.8%) and 21 (8.1%) from the second and first periods, respectively, died within 28 days from the onset of symptoms; and 39 (22.4%) and 30 (11.6%) from the second and first periods, respectively, died within 28 days from hospitalization. HR = hazard ratio; CI = confidence interval. This figure appears in color at www.ajtmh.org.

A total of 153 (87.9%) of 174 from the second and 157 (60.6%) of 259 from the first period required advanced respiratory support. The incidence risk of requiring advanced respiratory support was significantly higher in patients from the second than the first period (hazard ratio [HR]: 1.97; 95% confidence interval [CI]: 1.57–2.46; *P* < 0.001; Figure 2). The unadjusted risk for invasive respiratory support, 28-day mortality from onset of symptoms and from hospital admission were significantly higher in patients from the second than the first period (Figure 2). The adjusted risks for advanced respiratory support, invasive ventilatory support, and 28-day mortality from the onset of symptoms were significantly higher in patients from the second than the first period (Table 2). The risk for 28-day mortality from hospital admission (*P* = 0.09) was not statistically significant after adjustment (Table 2).

**Table 2:** Multivariate models for the advanced respiratory support, invasive ventilatory support, and 28-day mortality from the onset of symptoms and from hospitalization in patients from the Cohort 1

Variable	Adjusted hazard ratio (95% confidence interval)	*P*
Model 1: Advanced respiratory support from onset of symptoms*		
Second period†	2.04 (1.60–2.59)	< 0.001
Age	1.02 (1.00–1.03)	0.07
Sex, male	1.00 (0.79–1.26)	0.99
CCI score	0.99 (0.93–1.06)	0.81
BMI	1.03 (1.02–1.05)	< 0.001
Model 2: Invasive respiratory support from onset of symptoms‡		
Second period†	2.72 (2.05–3.62)	< 0.001
Age	1.03 (1.01–1.04)	< 0.001
Sex, male	0.97 (0.73–1.28)	0.83
CCI score	0.98 (0.91–1.07)	0.76
BMI	1.02 (1.00–1.03)	0.03
Model 3: 28-day mortality from onset of symptoms		
Second period†	2.62 (1.46–4.72)	0.001
Age	1.05 (1.02–1.08)	0.003
Sex, male	1.08 (0.61–1.93)	0.78
CCI score	1.14 (1.01–1.28)	0.04
BMI	1.00 (0.97–1.04)	0.83
Model 4: 28-day mortality from hospital admission		
Second period†	1.54 (0.93–2.57)	0.09
Age	1.03 (1.01–1.06)	0.008
Ordinal scale	1.18 (1.08–1.29)	< 0.001
CCI score	2.03 (1.59–2.60)	< 0.001

BMI = body mass index; CCI = Charlson’s Comorbidity Index.

* Advanced respiratory support was considered noninvasive ventilation, high-flow oxygen support, mechanical ventilation, or extracorporeal membrane oxygenation.

† Infections in patients from the second period are presumably caused by gamma.

‡ Invasive respiratory support was considered mechanical ventilation or extracorporeal membrane oxygenation.

In Cohort 2, a total of 72 (83.7%) gamma and 32 (74.4%) non-gamma patients required advanced respiratory support. The incidence rate of advanced respiratory support was significantly higher in gamma than in non-gamma patients (HR: 1.62; 95% CI: 1.01–2.57; *P* = 0.05 ( Supplemental Figure 1). The incidence rate of invasive respiratory support (*P* = 0.03) and death from onset of symptoms (*P* = 0.04) were significantly higher in gamma than in non-gamma patients ( Supplemental Figure 1), whereas the unadjusted incidence rate of death after hospitalization was notsignificantly higher (*P* = 0.10) in gamma than in non-gamma patients ( Supplemental Figure 1).

In the Cox proportional hazard model, gamma infection was associated with a higher risk of requiring advanced respiratory support ( Supplemental Table 2). The incidence rate of mechanical ventilation, death after 28 days from onset of symptoms and from hospital admission were also significantly higher in gamma- than in non-gamma-infected patients after adjustment for covariates (Table 2).

**Figure 3.**
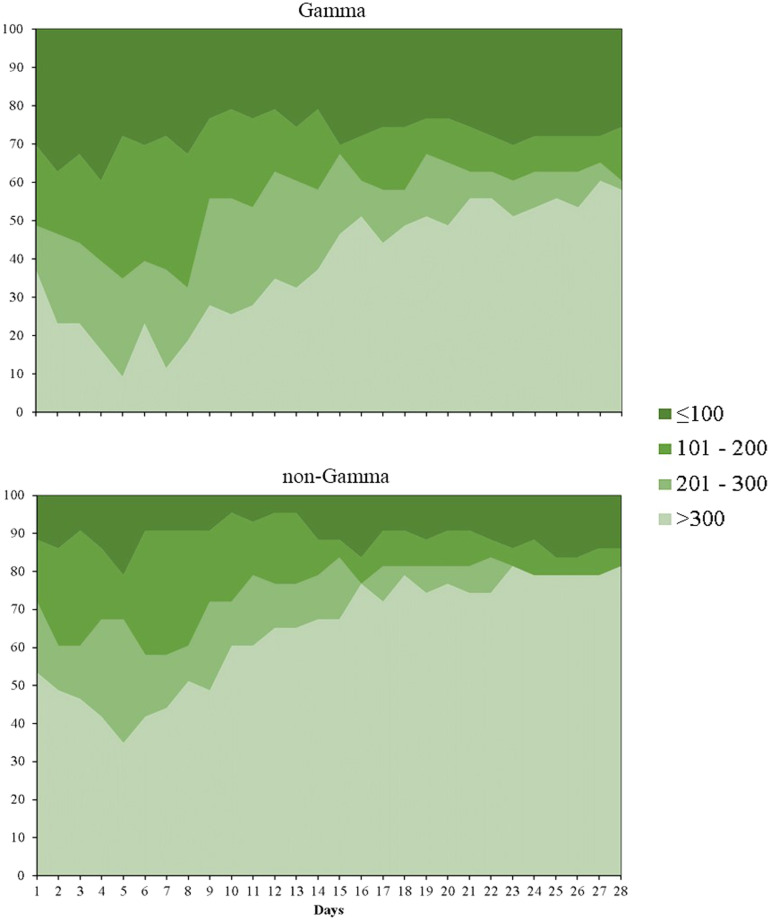
Pressure arterial oxygen/fraction of inspired oxygen ratio (PaO_2_/FiO_2_) in the first 28 days from hospitalization. This figure appears in color at www.ajtmh.org.

The status in the clinical ordinal scale and PaO_2_/FiO_2_ ratio on days 7, 14, 21, and 28 days in gamma and non-gamma groups of Cohort 2 are shown in Figure 3 and Supplemental Tables 5 and 6. Patients infected by gamma had significantly fewer days alive and free of supplemental oxygen support than those infected by non-gamma lineages (median: 2.0 days; interquartile range [IQR], 0.0–15.5 versus 18.0 days; IQR: 2.5–22.5; *P* < 0.001). There were no statistically significant differences between gamma and non-gamma patients in admission to ICU, need of renal replacement therapy, prone positioning, occurrence of thromboembolic event, and in-hospital mortality ( Supplemental Table 7). There was no significant difference in leukocyte counts, lymphocyte counts, C-reactive protein, and creatinine between groups along hospitalization ( Supplemental Figure 2).

## DISCUSSION

The collective findings of this retrospective cohort with non-elderly adult patients attending at the emergency department of a COVID-19 reference hospital suggest that infections occurred in the second period of the study (used as a proxy of gamma infections) presented a more severe course than those that occurred in the first period, before the emergence of gamma, as indicated by higher incidence rates of advanced and invasive respiratory support and death in the first 28 days from the onset of symptoms. Different from previous studies addressing the impact of VOC on hospitalization and death rates at the population level,^4–13^ our study assessed the progression of COVID-19 cause by gamma VOC and non-VOC lineages at the patient level. To the best of our knowledge, the comparison of the incidence of advanced ventilatory support in VOCs and non-VOCs has not been previously performed.

The results were similar in both cohorts—Cohort 1 in which infections occurred in the second and first periods of the study were presumably caused by gamma and non-gamma variants, respectively, and Cohort 2, which included randomly selected patients from Cohort 1 in whom infecting lineages were confirmed by WGS. The finding that patients from the second period presented to the emergency department with worse clinical status, as evidenced by higher scores in ordinal clinical scale and lower PaO_2_/FiO_2_ ratios, despite the similar time from onset of symptoms to admission observed in second and first period groups, corroborates the hypothesis that gamma infections may present a more severe course of the disease.

Advanced respiratory support was chosen as the primary outcome because during the gamma surge, there was a restriction in the number of ICU beds available^14,20^; thus, we a priori considered that differences in the incidence of mechanical ventilation, for example, could not be detected because invasive ventilatory support might be postponed due to limited access to ICU during this period. For instance, Bastos et al.^20^ have shown that the second wave in Brazil was associated with higher mortality among hospitalized patients but not with higher ICU admission, which might suggest a potential limitation in access to critical care. Surprisingly, in our study, the incidence rate of invasive respiratory support from the onset of symptoms was significantly higher in patients infected by gamma, even in the context of restricted access to ICU.

We included only non-elderly patients to minimize the effect of age on the course of the disease. An increase in the proportion of cases of COVID-19 in younger patients has been reported during the gamma surge in Brazil.^21^ In addition, we tried to minimize the effect of age on outcomes including this variable in the multivariate model.

Vaccination would be another factor that might affect the outcome; however, the vaccination of individuals who were 65 years or younger began in early April in our state; therefore, some patients of the second period could have received the first shot (mostly CoronaVac, Sinovac Biotech). Nonetheless, because we did not have these data standardly registered in medical records, we did not address this variable in our study. Despite this, vaccinated patients in the second group would decrease the effects of infections; thus, we believe it has not majorly affected the results.

In addition to those inherent to the retrospective design and those previously mentioned in the discussion, our study has other limitations that must be acknowledged. First, this is a single-center experience of a large tertiary-care COVID-19 reference hospital, which may restrict generalizability. Additionally, including only patients whose samples with Ct < 26 (criterion for sequencing adopted in this study due to limited number of WGS tests) may also affect generalizability and these results should be interpreted accordingly. Second, because gamma- and non-gamma-infected patients were hospitalized in different periods, they might be affected by distinct practices in COVID-19 management. However, it should be noted that more than 90% of patients were treated with corticosteroids (only assessed in the Cohort 2), and there was no other pharmacological intervention used in the second period. Furthermore, if better practices in COVID-19 patient care had been incorporated in more recent periods, it would favor the null hypothesis. Third, the study might be affected by a selection bias because the higher hospital occupancy in the second period may have delayed the admission of less severe cases. We cannot fully rule out the influence of selection bias, and it must be carefully considered in the interpretation of the results; however, time from onset of symptoms to hospital admission were essentially the same in both cohorts. Finally, our study addressed clinical evolution of patients with severe COVID-19 that required hospital admission and the finding cannot be extrapolated to outpatient population.

In summary, our study suggests that in nonelderly adult patients who require hospital admission by COVID-19 in the second period of the study—presumably caused by gamma—had worse outcomes, suggesting that gamma VOC might be associated with a more severe clinical course. In addition to the elevated number of cases resulting in ICU overload, increased severity of COVID-19 caused by gamma lineage may have contributed to the remarkably high ICU occupancy and death observed in the surge of this VOC in Brazil. The findings corroborate the hypothesis that clinical course of COVID-19 may differ among lineages, and this should be considering when estimating the impact of the emergence of distinct VOCs in the future.

## Supplemental Material


Supplemental materials
