# Supplementary material for: Evaluation of Clinical Course of Gamma (P.1) Variant of Concern versus Lineages in Hospitalized Patients with COVID-19 in a Reference Center in Brazil
Source: Am J Trop Med Hyg. 2022 Jun 27;107(2):245–51. doi: 10.4269/ajtmh.21-1264 (PMC9393469; doi:10.4269/ajtmh.21-1264)
Supplement: Supplementary file 1 [file tpmd211264.SD1.pdf]

# SUPPLEMENTARY METHODS

## Definitions and other prespecified secondary outcomes

Evolution in an ordinal clinical scale during the first 28 days was assessed by the proportion of patients in each category of an ordinal scale during the first 28 days (1, patient not hospitalized; 2, hospitalized and not receiving supplemental oxygen; 3, hospitalized and receiving supplemental oxygen; 4, hospitalized and receiving oxygen supplementation administered by non-invasive ventilation or high flow nasal cannula; 5, hospitalized and receiving mechanical ventilation or extracorporeal membrane oxygenation; and 6, death). The arterial partial pressure of oxygen/ fraction of inspired oxygen ratio (PaO2/FiO2 ratio during hospitalization, evaluated as the proportion of patients assigned in an ordinal scale according to this ratio (400-301, 300-201, 200-101, ≤100). For PaO2/FiO2 evaluation the worst value of the day was considered. Peripheral oxygen saturation (SpO2)/Fio2 corrected for positive end-expiratory pressure was used for days in which PaO2/FiO2 was not available (1).

Other outcomes assessed in Cohort 2 were days alive and free of supplemental oxygen support, defined as the number of days in which patients are alive and not receiving supplemental oxygen (non-survivors were assigned as 0 free-days); need of admission at an intensive care unit (ICU); occurrence of documented deep venous thrombosis or pulmonary embolism; need of renal replacement therapy (RRT); need of prone positioning; and in-hospital mortality.

### Real-time Reverse Transcription Polymerase Chain Reaction (RT-qPCR)

Oro/nasopharyngeal swabs were collected and submitted to RNA extraction followed by real-time reverse transcription-PCR (RT-qPCR) testing for two genes of the nucleocapsid protein (N1 and N2) of the SARS-CoV-2 as described by the Center for Disease Control and Prevention (2).

## Whole Genome Sequencing

All RT-qPCR positive clinical samples from patients attending at HCPA were stored at an institutional biobank. Sequencing libraries were prepared using the CleanPlex SARS-CoV-2 panel (Paragon Genomics. Hayward. United States) protocol for target enrichment and library preparation, following manufacturer instructions (https://www.paragongenomics.com/wp-content/uploads/2020/03/UG4001-01_-CleanPlex-SARS-CoV-2-Panel-User-Guide.pdf). The resulting libraries were sequenced in an Illumina MiSeq (Illumina. San Diego. US) equipment. Consensus sequences were generated by the QIASeq SARS-CoV-2 pipeline (QIAGEN CLC Genomics Workbench 21) with high quality (average coverage >350. <6% Ns. >29.8 Kb). The specimens were classified using the Phylogenetic Assignment of Named Global Outbreak Lineages (Pangolin) software tool (v3.1.5) and the sequences were deposited into the GISAID database (https://www.gisaid.org/).

### Sample Size

The sample size for the Cohort 2 was estimated based on the study of Ranzani et al. (3) which showed that the median time from onset of symptoms to hospital admission in Brazilian hospitals was 9 days in a period when variants of concern were not present. We estimated that patients who would require advanced respiratory support would require it in a median of 3 days. Therefore, the median number of days from onset of symptoms was estimated to be 12 in COVID-19 patients with COVID-19 caused by non-Gamma. For a 1:1 sample. an alpha of 0.05 and a beta of 0.20. estimating that populational standard deviation of 6 days (3) and aiming to detect a difference of at least 3 days for advanced respiratory support in Gamma-infected patients. the total sample size is 72 patients. The final sample size was defined as 86 patients, admitting a 20% increase in the sample since multivariable analysis is planned for this outcome.

### Complementary Statistical Procedures

The median number of days to the advanced respiratory support was evaluated by Mann-Whitney test. For this later analysis, the number of days for patients who have not required advanced respiratory support was considered undetermined. Secondary outcomes were analyzed using chi-square or Fisher's exact test. Mann-Whitney, log-rank and ordinal logistic regression.

For 28-day mortality from hospitalization, a Cox regression model was constructed including age (regardless of the P value) and variables with a P value ≤0.20 in the bivariate analysis, using forward stepwise selection. Variables with a P value ≤0.05 were maintained in the model. A gamma generalized estimating equations model with Bonferroni correction was used to simultaneously assess the parameters over time and between groups.

## SUPPLEMENTARY RESULTS

**STable 1** Summary of SARS-CoV-2 lineages causing infection in patients included in the Cohort 2.

| **Period** | **Lineage** | **n (%)** |
|---|---|---|
| First: June to December 2020 | | 42 |
| | B.1.1.28 | 15 (37.5) |
| | B.1.1.161 | 10 (23.8) |
| | B.1.1 | 5 (11.9) |
| | B.1.1.33 | 3 (7.1) |
| | B.1.28 | 2 (4.8) |
| | P.2 | 2 (4.8) |
| | B.1.1.370 | 1 (2.4) |
| | B.1.1.409 | 1 (2.4) |
| | B.1.1.462 | 1 (2.4) |
| | B.1.91 | 1 (2.4) |
| | P.1 | 1 (2.4) |
| Second: February to May 2021 | | 44 |
| | P.1 | 38 (86.4) |
| | P.1.1 | 2 (2.3%) |
| | P.1.2 | 2 (2.3%) |
| | P.2 | 2 (2.3%) |

**Stable 2.** Baseline characteristics of patients from the Cohort 2.

| Characteristics | Total (n=86) | Gamma (n=43) | non-Gamma (n=43) | P value |
|---|---|---|---|---|
| Gender, male | 46 (53.5) | 26 (60.5) | 20 (46.5) | 0.28 |
| Age, years | 51.0 (43.5 - 59.0) | 51.0 (39.0 - 58.0) | 51.0 (46.0 - 61.0) | 0.39 |
| Charlson's Comorbidity Score | 1.0 (0.0 - 3.0) | 0.0 (0.0 - 2.0) | 2.0 (0.0 - 3.0) | 0.04 |
| Comorbidities | | | | |
| Diabetes | 22 (25.6) | 9 (20.9) | 13 (30.2) | 0.46 |
| Hypertension | 47 (54.7) | 20 (46.5) | 27 (62.8) | 0.19 |
| Cardiovascular Disease | 15 (17.4) | 5 (11.6) | 10 (23.3) | 0.26 |
| Chronic Pulmonary Disease | 4 (4.7) | 2 (4.7) | 2 (4.7) | 0.99 |
| BMI, $kg/m^2$ [a] | 31.0 (27.3 - 36.9) | 30.8 (27.6 - 36.9) | 31.2 (28.0 - 37.0) | 0.65 |
| BMI ≥30 $kg/m^2$ [a] | 51 (63.8) | 26 (63.4) | 25 (64.1) | 0.99 |
| Time from onset of symptoms to hospital admission, days | 7.0 (4.3 - 9.0) | 7.0 (4.0 - 8.0) | 7.0 (5.0 - 9.0) | 0.67 |
| NEWS 2 | 7.0 (4.0 - 9.0) | 7.0 (5.5 - 8.0) | 6.0 (3.0 - 9.0) | 0.09 |
| PaO2/FiO2 at admission | | | | |
| >300 | 39 (45.3) | 16 (37.2) | 23 (53.5) | 0.12 |
| 300-201 | 13 (15.1) | 5 (11.6) | 8 (18.6) | |
| 200-101 | 16 (18.6) | 9 (20.9) | 7 (16.3) | |
| ≤100 | 18 (20.9) | 13 (30.2) | 5 (11.6) | |
| Score on six-level ordinal scale | | | | |
| 2- hospitalization without supplemental oxygen | 30 (34.9) | 11 (25.6) | 19 (44.2) | 0.21 |
| 3 - hospitalization with supplemental oxygen | 41 (47.7) | 22 (51.2) | 19 (44.2) | |
| 4 - hospitalization with non-invasive ventilation or high-flow supplemental oxygen | 8 (9.3) | 6 (14.0) | 2 (4.7) | |
| 5 - hospitalization with invasive mechanical ventilation and/or extracorporeal membrane oxygenation | 7 (8.1) | 4 (9.3) | 3 (7.0) | |
| Oxygen delivery device | | | | |
| None | 30 (34.9) | 11 (25.6) | 19 (44.2) | 0.48 |
| Low-flow nasal cannula | 19 (22.1) | 10 (23.3) | 9 (20.9) | |
| Hudson mask | 22 (25.6) | 12 (27.9) | 10 (23.3) | |

| | | | | |
|---|---|---|---|---|
| Non-invasive ventilator | 4 (4.7) | 3 (7.0) | 1 (2.3) | |
| High flow nasal cannula | 4 (4.7) | 3 (7.0) | 1 (2.3) | |
| Invasive mechanical ventilator | 7 (8.1) | 4 (9.3) | 3 (7.0) | |
| Laboratorial findings | | | | |
| Ct, N1 | 20.35 ± 3.24 | 19.74 ± 3.35 | 20.96 ± 3.05 | 0.08 |
| Ct, N2 | 20.58 ± 3.42 | 20.30 ± 3.73 | 20.86 ± 3.09 | 0.45 |
| White blood cell count, cells $\times 10^3/\mu L$ [b] | 7.2 (5.4 - 10.6) | 7.0 (5.2 - 10.6) | 8.0 (5.5 - 10.9) | 0.72 |
| Neutrophil count, cells $\times 10^3/\mu L$ [b] | 5.7 (4.1 - 8.5) | 5.6 (4.0 - 9.3) | 6.2 (4.2 - 8.5) | 0.71 |
| Lymphocyte count, cells/μL [b] | 826.1 (637.6 - 1211.8) | 770.0 (563.7 - 1004.0) | 870.4 (676.5 - 1239.8) | 0.13 |
| Platelet count, cells $\times 10^3/\mu L$ [b] | 205.5 (155.0 - 265.0) | 181.0 (142.0 - 226.0) | 246.0 (174.0 - 279.5) | 0.01 |
| C reactive protein, mg/L [c] | 124.5 (75.6 - 202.0) | 123.6 (74.2 - 191.1) | 126.8 (83.1 - 205.0) | 0.62 |
| D-dimer, μg/mL [d] | 0.8 (0.5 - 1.4) | 0.8 (0.5 - 1.4) | 0.9 (0.4 - 1.4) | 0.99 |
| Serum creatinine, mg/dL [e] | 0.9 (0.8 - 1.4) | 0.9 (0.8 - 1.4) | 1.0 (0.8 - 1.3) | 0.78 |

Data expressed as n (%), median (IQR) or mean ± SD. BMI, body mass index; PaO2/FiO2, partial pressure of arterial oxygen /fractional inspired oxygen; NEWS, National Early Warning Score; Ct, cycle threshold.

[a] Two (4.7%) patients in P.1 and 4 (9.3%) in non-P.1.did not have BMI recorded.

[b] One (1.2%) patient from P.1 group did not have white blood cells recorded; and one (2.3%) patient in P.1 did not have platelets recorded.

[c] Two (4.7%) in P.1 and 3 (7.0%) in non-P.1 did not have C reactive protein recorded.

[d] One (1.2%) patient from P.1 group did not have D-dimers recorded.

[2] Three (7.0%) patients from non-P.1 did not have creatinine recorded.

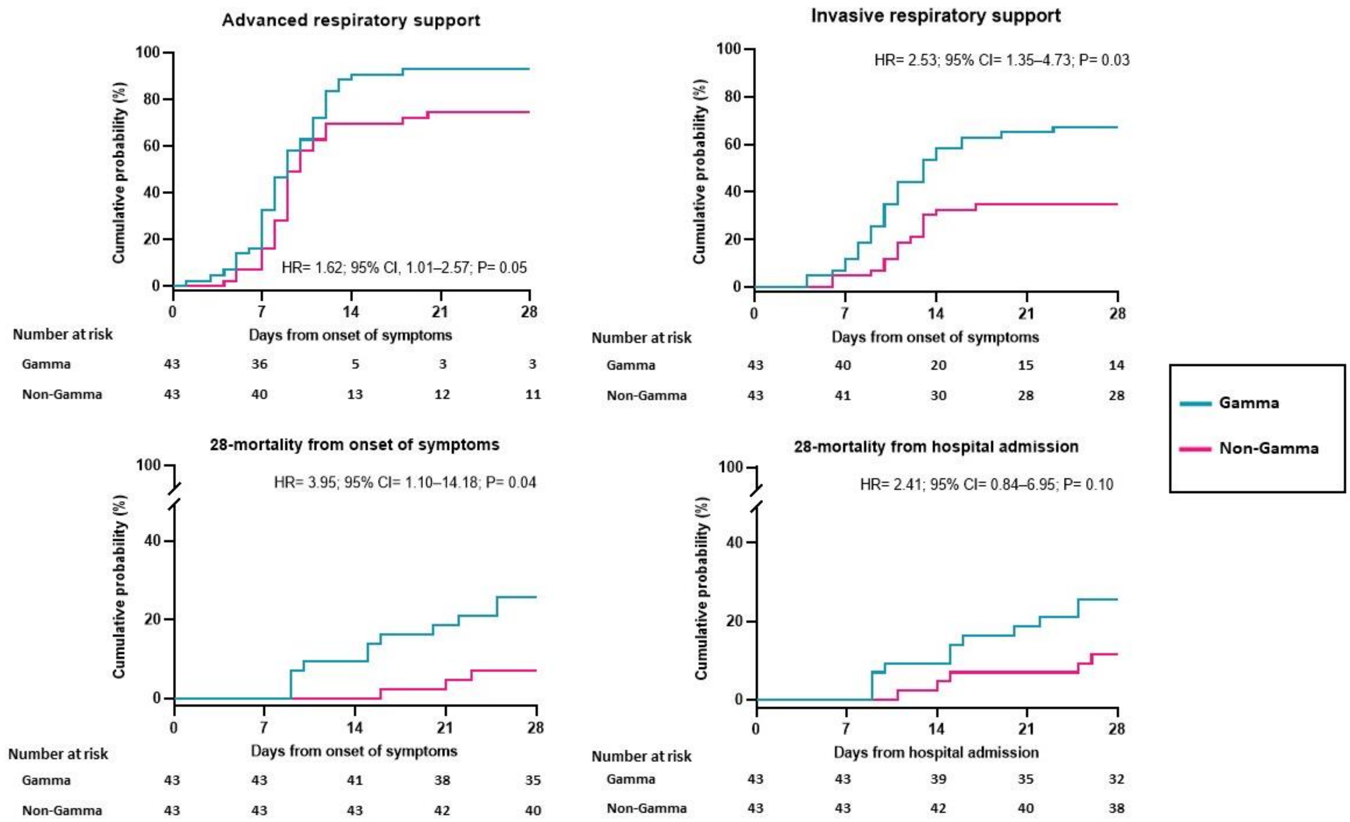


**SFigure 1.** Primary and major secondary outcomes in the Cohort 2.

Twenty-nine (67.4%) and 14 (32.6%) Gamma and non-Gamma patients, respectively, required invasive respiratory support ; 11 (25.6%) Gamma and 3 (7.0%) non-Gamma patients died in the first 28 days from the onset of symptoms; 11 (25.6%) and 5 (11.6%) Gamma and non-Gamma patients, respectively, died in the first 28 days of hospitalization.

**STable 4.** Multivariate models for the advanced respiratory support, invasive ventilatory support, and 28-day mortality from the onset of symptoms and from hospitalization in Cohort 2.

| Variable | Hazard ratio | 95% Confidence Interval | P |
|---|---|---|---|
| **Model 1: Advanced respiratory support from onset of symptoms** [a] | | | |
| Gamma infection | 1.78 | 1.05 - 3.03 | 0.03 |
| Age | 1.01 | 0.99 - 1.03 | 0.56 |
| Sex, male | 1.24 | 0.76 - 2.03 | 0.40 |
| Charlson's Score | 1.00 | 0.91 - 1.10 | 0.93 |
| Body Mass Index | 1.04 | 1.02 - 1.07 | 0.02 |
| **Model 2: Invasive respiratory support from onset of symptoms** [b] | | | |
| Gamma infection | 2.64 | 1.34 - 5.19 | 0.005 |
| Age | 1.02 | 0.99 - 1.05 | 0.17 |
| Sex, male | 1.17 | 0,62 - 2.21 | 0.62 |
| Charlson's Score | 1.04 | 0.93 - 1.16 | 0.53 |
| Body Mass Index | 1.01 | 0.97 - 1.04 | 0.64 |
| **Model 3: 28-day mortality from onset of symptoms** | | | |
| Gamma infection | 4.73 | 1.15 - 19.41 | 0.03 |
| Age | 1.04 | 0.98 - 1.10 | 0.18 |
| Sex, male | 1.13 | 0.35 - 3.61 | 0.84 |
| Charlson's Score | 1.14 | 0.94 - 1.39 | 0.20 |
| Body Mass Index | 0.97 | 0.88 - 1.06 | 0.45 |
| **Model 4: 28-day mortality from hospital admission** [c] | | | |

| | | | |
|---|---|---|---|
| Gamma infection | 3.72 | 1.19 - 11.65 | 0.02 |
| Age | 1.02 | 0.97 - 1.07 | 0.45 |
| Charlson`s Score | 1.21 | 1.05 - 1.38 | 0.007 |

[a] Advanced respiratory support was considered non-invasive ventilation, high-flow oxygen support, mechanical ventilation or extracorporeal membrane oxygenation.

[b] Invasive respiratory support was considered mechanical ventilation or extracorporeal membrane oxygenation.

[c] Forcing age into the model did not modify the effect of Gamma lineage on the outcome.

**STable 5.** Risk of Gamma-infected patients in comparison to non-Gamma-infected patients of receiving oxygen supplementation by non-invasive or invasive methods at the baseline and in four weeks of follow-up after hospitalization.

| | Odds Ratio (95% Confidence Interval) for Gamma infections | | | | |
|---|---|---|---|---|---|
| Ordinal Scale Category [a] | Baseline | Day 7 | Day 14 | Day 21 | Day 28 |
| 1 or 2 | reference | reference | reference | reference | reference |
| 3 or 4 [b] | 2.30 (0.91-5.86) | 3.86 (0.75-20.01) | 2.16 (0.66-7.02) | 0.60 (0.16- 2.21) | 0.762 (0.28-4.54) |
| 5 or 6 [b] | 2.30 (0.43-12.25) | 10.91 (2.04-58.39) | 3.51 (1.31-9.47) | 5.25 (1.77-15.55) | 4.35 (1.56-12.09) |
| P [c] | 0.19 | 0.003 | 0.04 | 0.001 | 0.009 |

[a] Category 1, non-hospitalized without supplemental oxygen; 2, hospitalization without supplemental oxygen; 3, hospitalization with low-flow supplemental oxygen; 4, hospitalization with non-invasive ventilation or high-flow supplemental oxygen; 5, hospitalization with invasive mechanical ventilation and/or extracorporeal membrane oxygenation; 6, Death.

[b] The odds ratio refers to category 1 or 2 (reference).

[c] Obtained by ordinal logistic regression model.

**STable 6.** Risk of Gamma-infected patients in comparison to non-Gamma-infected patients of having a reduced pressure arterial oxygen/ fraction of inspired oxygen ratio (PaO2/FiO2) at the baseline and in four weeks of follow-up after hospitalization.

| | Odds Ratio (95% Confidence Interval) | | | | |
|---|---|---|---|---|---|
| PaO2/FiO2[a] | Baseline | Day 7 | Day 14 | Day 21 | Day 28 |
| >300 | reference | reference | reference | reference | reference |
| 300-201 [b] | 0.90 (0.25-3.25) | 6.98 (1.72-28.25) | 3.26 (0.93-11.41) | 1.33 (0.25-7.19) | [c] |
| 200-101 [b] | 1.85 (0.57-5.99) | 4.07 (1.20-13.86) | 4.08 (1.08-15.37) | 1.67 (0.40- 6.88) | 4.20 (0.78- 22.55) |
| ≤100 [b] | 3.74 (1.11-12.57) | 11.40 (2.54-51.11) | 3.26 (0.93- 11.41) | 3.67 (1.04-12.94) | 2.57 (0.84-7.86) |
| P. [c] | 0.12 | 0.002 | 0.04 | 0.20 | |

[a] >300 corresponds to no Acute Distress Respiratory Syndrome (ARDS); 300-201, mild ARDS; 200-101, moderate ARDS; ≤100, severe ARDS.

[b] The odds ratio refers to category 1 or 2 (reference).

[c] The risk was not determined since there was no non-Gamma patient at this category on day 28.

[d] Obtained by ordinal logistic regression model.

**STable 7.** Other secondary outcomes in Cohort 2.

| Outcome | Gamma<br>n (%) | non-Gamma<br>n (%) | Relative Risk<br>(95% CI) | P |
|---|---|---|---|---|
| Admission at ICU | 29 (67.4) | 20 (46.5) | 1.45 (0.99-2.12) | 0.08 |
| Need of prone positioning | 8 (18.6) | 8 (18.6) | 1.00 (0.41-2.42) | 0.99 |
| Need of RRT | 14 (32.6) | 11 (25.6) | 1.27 (0.65-2.48) | 0.64 |
| Occurrence of thromboembolic event | 7 (16.3) | 6 (14.0) | 1.17 (0.43-3.19) | 0.99 |
| In-hospital mortality. n (%) | 12 (27.9) | 8 (18.6) | 1.28 (0.82-1.94) | 0.44 |

CI, Confidence Interval; ICU, Intensive Care Unit; RRT, Renal Replacement Therapy.

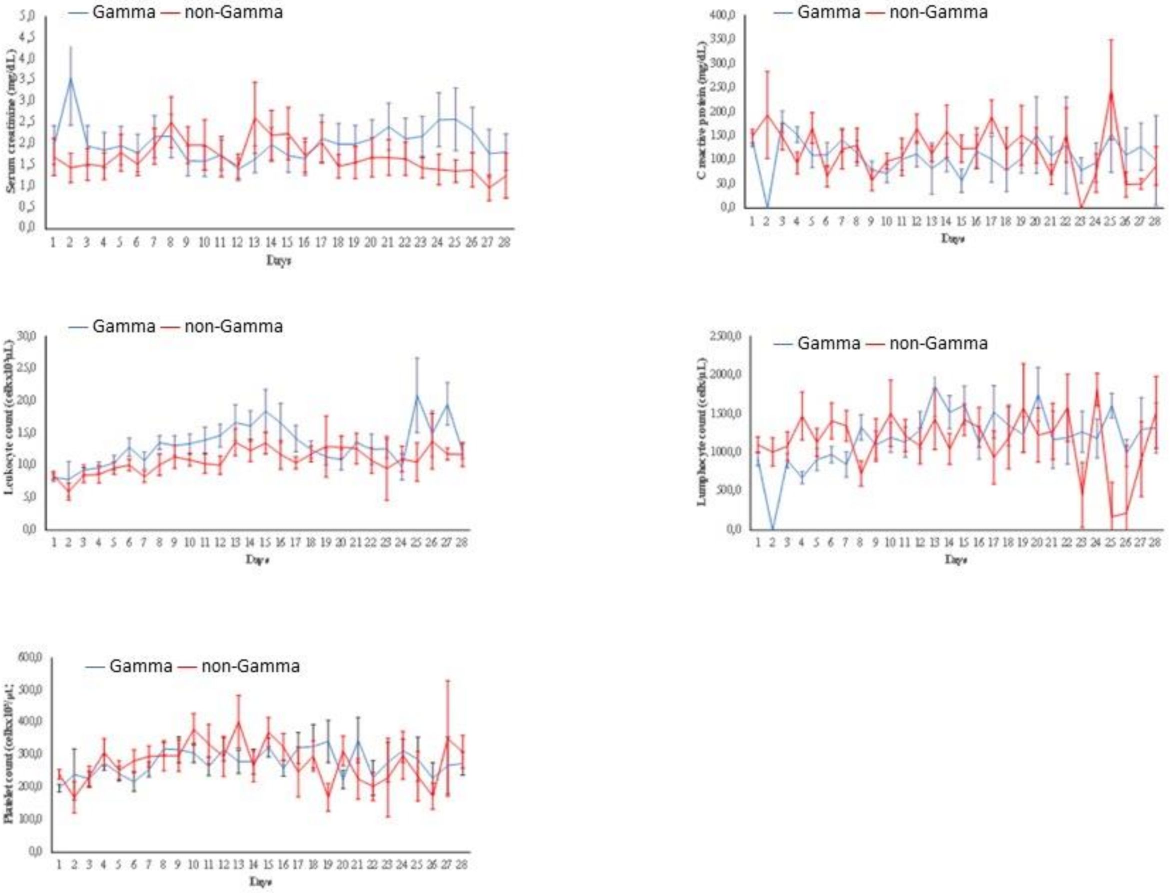


**SFigure 2.** Laboratorial exams from baseline to day 28 of hospitalization.

Generalized estimating equations indicated no effect of variant on C reactive protein (P=0.35), creatinine (P=0.65), leukocyte count (P=0.34) and lymphocyte count (P=0.46) along the 28-day period.

## References

1. Pandharipande PP, Shintani AK, Hagerman HE, St Jacques PJ, Rice TW, Sanders NW, et al. Derivation and validation of Spo2/Fio2 ratio to impute for Pao2/Fio2 ratio in the respiratory component of the Sequential Organ Failure Assessment score. Crit Care Med. 2009;37(4):1317-21.
2. Prevention. CDC. Information for laboratories. 2019 novel coronavirus, Wuhan, China. 2020 [Available from: https://www.cdc.gov/coronavirus/2019-nCoV/guidance-laboratories.html.
3. Ranzani OT, Bastos LSL, Gelli JGM, Marchesi JF, Baião F, Hamacher S, et al. Characterisation of the first 250,000 hospital admissions for COVID-19 in Brazil: a retrospective analysis of nationwide data. Lancet Respir Med. 2021;9(4):407-18.
